# Baduanjin exercise: A potential promising therapy toward osteoporosis

**DOI:** 10.3389/fmed.2022.935961

**Published:** 2022-08-03

**Authors:** Chuanrui Sun, Baoyu Qi, Xinyi Huang, Ming Chen, Zikai Jin, Yili Zhang, Liguo Zhu, Xu Wei

**Affiliations:** ^1^Wangjing Hospital, China Academy of Chinese Medical Sciences, Beijing, China; ^2^School of Tradition Chinese Medicine, Beijing University of Chinese Medicine, Beijing, China; ^3^School of Traditional Chinese Medicine and School of Integrated Chinese and Western Medicine, Nanjing University of Chinese Medicine, Nanjing, China

**Keywords:** Baduanjin exercise, Chinese qigong, osteoporosis, evidence-based medicine, meta-analysis, trial sequential analysis

## Abstract

**Purpose:**

Baduanjin (BDJ) exercise is a traditional exercise that combines breathing, body movement, meditation and awareness to help delay the onset and progression of senile degenerative musculoskeletal diseases, such as osteoporosis (OP). The aim of this meta-analysis is to evaluate the efficacy of BDJ exercise, and preliminarily infer its effective mechanism in the treatment of OP.

**Methods:**

We identified relevant randomized controlled trials (RCTs) through eight databases, and compared BDJ exercise with the control groups (including blank control and conventional treatment intervention). The main outcome measure was bone mineral density (BMD), the additional outcome measures were visual analogue scale (VAS), Berg balance scale (BBS), serum Calcium (Ca), serum Phosphorus (P), serum Alkaline phosphatase (ALP), and serum bone gla protein (BGP). Meta-analysis and trial sequence analysis (TSA) were performed using RevMan 5.4, Stata 16.0, and TSA 0.9.

**Results:**

In total, 13 RCTs involving 919 patients were included in the analysis. For postmenopausal osteoporosis, BDJ exercise alone and BDJ exercise combined with conventional treatment can improve the BMD of lumbar spine. BDJ exercise alone can influence serum Ca and ALP. BDJ exercise combined with conventional treatment can improve balance (BBS) and influence serum BGP. For senile osteoporosis, BDJ exercise alone and BDJ exercise combined with conventional treatment can improve balance (BBS). BDJ exercise combined with conventional treatment can improve the BMD of hip and pain relieve (VAS). For primary osteoporosis, BDJ exercise combined with conventional treatment can improve the BMD of lumbar spine and femoral neck.

**Conclusion:**

Baduanjin exercise may be beneficial to improve BMD, relieve pain, improve balance ability, influence serum BGP and serum ALP in patients with OP, but differences occur due to various types of OP. Due to the low quality of research on the efficacy and mechanism of BDJ exercise in the treatment of OP, high-quality evidence-based research is still needed to provide reliable supporting evidence.

**Systematic Review Registration:**

[http://www.crd.york.ac.uk/PROSPERO], identifier [CRD42022329022].

## Introduction

Osteoporosis (OP) is a common metabolic disease closely related to population aging, but it is often overlooked by people until symptoms appear, such as unexplained pain and fractures (1). With global population aging, OP has become a heavy public health problem, placing a heavy burden on families and health care systems. The global prevalence of OP in the elderly is 21.7%, and respectively, the prevalence rates in Asia, Europe, and the United States are 24.3, 16.7, and 11.5%, with the highest prevalence in Asia (2). In addition, the economic burden caused by OP is exceedingly heavy. Take China as an example, by 2035, the annual number and cost of OP, along with related fractures will double, and the cost is about 25.4 billion dollars by 2050 (3). Population aging is inevitable, but healthy aging is an issue that we can touch (4), so we should make more efforts to solve this problem.

At present, the clinical treatments of OP mainly use bone resorption inhibitor drugs and bone formation promoter drugs, but there are some inevitable adverse reactions (5, 6). In addition, although pharmacological strategies for OP have been shown to be highly effective, only a small number of patients receive treatments (7), so it’s required to find a potential alternative therapy with few side effects and easy promotion, which is very significant and valuable.

Baduanjin (BDJ) is a health-preserving exercise recommended by the *Chinese Health Qigong Association* (8). It is an important exercise form of ancient *Chinese Qigong*, especially for the prevention and rehabilitation of diseases. There is abundant evidence that regular exercise can improve overall health and protect against many adverse health risk factors (9–11). At the same time, there is a close relationship between exercise and bone health, and exercise is widely recommended to improve bone health (12). Exercise also has outstanding advantages in the treatment of OP. A study has shown that resistance and weight-bearing exercise can increase muscle mass and temporarily increase BMD (13). Some scholars have reviewed that different modes of exercise have a positive effect on fractures, falls, and bone mass in the elderly (14). Meanwhile, lifelong exercise (including weight-bearing exercise and muscle strengthening exercise) can prevent OP (15). It is delightful that all eight movements of BDJ exercise involve resistance exercise and weight-bearing exercise.

The number of clinical trials related to BDJ exercise for OP is increasing every year, however, these have not been fully summarized according to the latest evidence. Therefore, we conduct an updated meta-analysis of randomized controlled trials (RCTs) to evaluate the efficacy of BDJ exercise in patients with OP, and initially summarized potential mechanism of BDJ exercise at the same time, which provides a reference for clinical practice and scientific research.

## Methods

### Data Sources and Search Strategies

We searched^1^ four English electronic databases and four Chinese electronic databases, including Medline, Cochrane Library, EMBASE, Web of Science, China National Knowledge Infrastructure (CNKI), Wang Fang, China Science and Technology Journal Database (VIP), and Chinese Biomedical Literature Database (CBM), from the date of establishment to April 2022, without language restrictions. A systematic search was carried out by three researchers (Chuanrui Sun, Baoyu Qi, and Yili Zhang), and any discrepancies between researchers were agreed through discussion. To find other relevant studies, all potential articles were also searched and discussed.

### Study selection and eligibility

#### Types of studies

All included studies were RCTs, and not restricted by publication. The cross-sectional studies, animal experiments, systematic reviews and meta-analyses, disease guidelines, and studies of which we did not have access to the full texts were excluded.

#### Types of participants

We have always focused on the effect of BDJ exercise on BMD. First of all, the disease we were concerned about was OP, and the clinical diagnosis should conform to recognized international standards. In WHO: T-score ≤ −2.5 can be defined as OP. OP includes two types, primary osteoporosis (POP) and secondary osteoporosis. POP is further divided into postmenopausal osteoporosis (PMOP, type I), senile osteoporosis (SOP, type II) and idiopathic osteoporosis (including juvenile osteoporosis). When we screened the studies, we also found that some studies mentioned “diabetic OP” and “psoriatic OP.” After discussion, we thought these situations should be excluded in this study. Ultimately, we focused our attention on POP as the subject of our study.

#### Types of interventions

In order to evaluate the independent effect of BDJ exercise, we included the studies in which the intervention method was only BDJ exercise. In addition, we also took another situation into account, that was, when the control group was the conventional treatment, for example, Calcium Carbonate Chewable D3 tablet, et al., the intervention group was BDJ exercise combined with conventional treatment. In this case, the independent effect of BDJ exercise could also be observed. In addition, we had no restrictions on the teaching method, exercise frequency, and intervention time of BDJ exercise.

#### Types of comparisons

In the control group, we included only two cases: (A) no treatment; (B) conventional treatment: guideline-recommended routine treatments or internationally recognized treatments. Calcium and Vitamin D supplementation was also considered conventional treatment. Other combined treatments or alternative treatments were excluded because it was impossible to determine whether BDJ exercise played an independent role, such as drugs that had not been verified for efficacy with high evidence, including Chinese herbal decoctions, Chinese herbal medicated meals, etc.

#### Types of outcomes

Bone mineral density (BMD) in patients with OP was the primary outcome we observed. The additional outcomes included visual analogue scale (VAS), Berg balance scale (BBS), serum Calcium (Ca), serum Phosphorus (P), serum Alkaline phosphatase (ALP), serum bone gla protein (BGP).

### Data extraction

Three members (CS, XH, and BQ) independently screened and extracted data, and disagreements were handled by discussion or consensus with other members. The following data were extracted: first author, country, OP types, sample size, gender, intervention, frequency and duration, outcome measures.

### Quality assessment

The quality assessment was conducted independently by three researchers (MC, ZJ, and XW), and differences between researchers were resolved through discussion. Risk of bias assessments for all included RCTs were described according to the *Cochrane Handbook for Systematic Reviews of Interventions*. Criteria mainly included: (A) Random sequence generation; (B) Allocation concealment design treatment; (C) Participant and rater blindness; (D) Withdrawal and loss of follow-up; (E) Incomplete outcome data and selective outcome reporting; (F) Other bias. Evaluation methods were high risk of bias, low risk of bias, and unknown risk of bias.

### Statistical methods

Analyses were performed using Cochrane Collaboration software (RevMan Version 5.3, Copenhagen, The Nordic Cochrane Centre) and Stata 16.0 (Texas, Stata Corp.). Continuous data use weighted mean difference (WMD) or STD Mean Difference (SMD). Meanwhile, according to the heterogeneity test, *I*^2^ test statistic were used for evaluation. If there was no serious heterogeneity (I^2^ ≤ 50%), a fixed-effects model was selected for meta-analysis, otherwise, a random-effects model was used. Trial sequence analysis (TSA) were performed using TSA 0.9.

## Results

### Study selection

**FIGURE 1:**
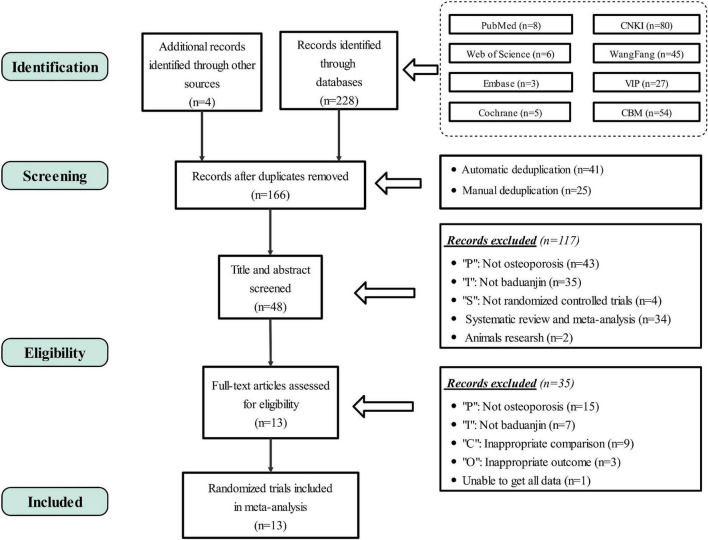
PRISMA flow diagram. PRISMA, preferred reporting items for systematic review and meta-analysis; RCTs, randomized controlled trials; P, population; I, intervention; C, comparison; O, outcome.

In total, we collected 232 records, including 228 records that we retrieved from eight common databases, and 4 RCTs that we traced back through research by others. Combined the automatic deduplication (*n* = 41, Endnote, Thomson ResearchSoft) with manual deduplication (*n* = 25), we removed duplicate documents and left 166 records. Then, we excluded 117 records that didn’t meet the criteria by reading the title or abstract. At last, remaining 48 records required us to read the full text and careful evaluate, and 35 articles were further excluded. Finally, a total of 13 records were included in our study, including one in English and 12 in Chinese. The detailed process of search and identification is shown in Figure 1.

### Study characteristics

There were 13 trials with a parallel randomized control design. Trial durations ranged from 3 to 12 months, with most 6 or 12 months. A total of 919 people were included in this review, including 462 in the experimental group and 457 in the control group. All included trials reported results of our interest, 12 of which involved BMD values (primary outcome measure). Finally, characteristic details of the 13 included trials were listed (Table 1).

**TABLE 1:** Characteristics of included trials.

Study ID	Country	Types	Size		Gender		Intervention		Intervention details		Outcomes
			C	T	M	F	C	T	C	T	
Wang and Sha (75)	China	PMOP	36	36	/	72	Blank	BDJ exercise	None	50 min each time, twice a day, 5 days a week for 3 months	(1) BMD (L1–L4 and femoral neck) (2) BGP/ALP/U-HOP/U-Ca/U-Cr
Miao and Wang (76)	China	PMOP	12	12	/	24	Blank	BDJ exercise	None	60 min each time, once a day, 6 days a week for 12 months	(1) BMD (L2, Distal 1/3 of ulna and radius) (2) Ca/P/ALP/DPD/Cr
Li (77)	China	PMOP	25	21	/	46	Blank	BDJ exercise	None	70 min each time, five times a week for 24 weeks	(1) BMD (L2–L4 and dominant proximal femur)
Cai et al. (78)	China	PMOP	30	30	/	60	Conventional treatment	Conventional treatment + BDJ exercise	Calcium carbonate and vitamin D3 tablets (one time a day, 0.6 g each time for 12 months)	Twice per day, 5 days a week for 6 months	(1) BMD (L2-L4) (2) Ca/P/ALP (3) Quality of life score
Su and Deng (79)	China	PMOP	40	40	/	80	Conventional treatment	Conventional treatment + BDJ exercise	(1) Alendronate sodium (70 mg/time, 1 time/week) (2) Calcium D3 (0.6 g/time, 1 time/day) (3) Calcitriol capsules (0.25 μg/time, 1 time/day) Intervention for 6 months	45–60 min each time, twice per day, 5 days a week for 6 months	(1) BMD (L2–L4) (2) BGP/E2; DPD, Cr (3) TUG (Time up and go test) (4) BBS (Berg balance scale) (5) VAS
Zhang et al. (80)	China	PMOP	36	36	/	72	Conventional treatment	Conventional treatment + BDJ exercise	Calcium carbonate and vitamin D3 Tablets (one time a day, 0.6 g each time for 12 months)	Twice per day, 5 days a week for 12 months	(1) BMD (L2–L4 and left femoral neck, the baseline, the 12 weeks and 24 weeks) (2) Ca/P/ALP
Chen et al. (81)	China	PMOP	43	44	/	87	Blank	BDJ exercise	None	Learn for 2 weeks, 3 days a week for 12 weeks	(1) BMD (2) IL-6
Liu (82)	China	POP	30	30	31	29	Conventional treatment	Conventional treatment + BDJ exercise	Such as calcium preparations, etc.	60 min per day, 6 days a week for 6 months	(1) BMD (2) Bone pain score
Li et al. (83)	China	SOP	35	38	/	/	Blank	BDJ targeted exercise	Simply distributing fall prevention health education brochures	Five times a week, including twice targeted exercise, three times independent exercise for 24 weeks	(1) BMD (L2-L4 and double hip, the baseline, 12th weeks and the 24th weeks) (2) BBS (Berg balance scale, the baseline, 12th weeks and the 24th weeks)
Li et al. (83)	China	SOP	35	37	/	/	Blank	BDJ independent exercise	Simply distributing fall prevention health education brochures	Distributing BDJ exercise CDs and practice wall charts	(1) BMD (L2–L4 and double hip, the baseline, 12th weeks and the 24th weeks) (2) BBS (Berg balance scale, the baseline, 12th weeks and the 24th weeks)
Sun et al. (84)	China	SOP	30	30	15	45	Conventional treatment	Conventional treatment + BDJ exercise	Alfacalcidol (0.5 μg/day), calcium (0.6 g/day), alendronate (70 mg/week)	50 min each time, three times a week for 12 months	(1) BMD (L2–L4, femoral neck and double hip) (2) SPPB (Short physical performance battery) (3) MFES (Modified falls efficacy scale) (4) VAS (5) SF-36 (the short-form 36 item health survey questionnaire)
Kuang (85)	China	SOP	41	41	/	/	Conventional treatment	Conventional treatment + BDJ exercise	Calcium carbonate and vitamin D3 Tablets (two time a day, one tablets each time for 6 months)	60 min each time, twice daily for 3 months	(1) BMD (L2-L4, femoral neck and double hip) (2) BBS (Berg balance scale) (3) VAS (4) SF-36
Sun et al. (86)	China	SOP (male)	25	18	43	/	Conventional treatment	Conventional treatment + BDJ exercise	Calcium, vitamin D, alendronate and salmon calcitonin. Intervention for 6 months	More than 20 times in 4 weeks for 6 months	(1) BMD (L1-L4 and femoral neck) (2) VAS (the baseline and every other month during treatment) (3) ODI (Oswestry disability index)
Li et al. (87)	China	SOP	44	44	28	60	Conventional treatment	Conventional treatment + BDJ exercise	Calcium carbonate and vitamin D3 Tablets and salmon calcitonin	30–40 min each time, once daily for 6 months	(1) ECLSB (Eyes closed and single legged standing balance capacity) (2) TUGT (Timed up and go test) (3) BBS (Berg Balance Scale) (4) MFS (Morse Fall Scale) (5) SAS (Self-Rating Anxiety Scale)

C, control group; T, treatment group; M, male; F, female; PMOP, postmenopausal osteoporosis; SOP, senile osteoporosis; POP, primary osteoporosis.

### Methodological quality

**FIGURE 2:**
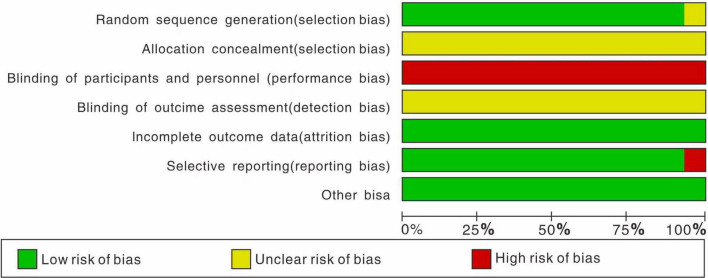
Risk of bias graph.

All trials were randomized, most of them using random number tables. It’s a pity that no trials mentioned allocation concealment, due to the particularity of BDJ exercise. It’s worthy noting that, however, in trials that use exercise as an intervention, blinding is difficult to conduct. We found no relevant protocols on the ClinicalTrials.gov website. In addition, six trials reported subject withdrawal, and only one trial reported in detail the cause, such as pain, constipation, fracture, etc. In summary, our evaluation of the RCTs quality was presented in Figure 2.

### The effects of therapy

Through preliminary analysis, we found that BDJ exercise had a positive effect on BMD, pain and balance in patients with OP, mainly manifested in lumbar spine BMD, femoral neck BMD, VAS, BBS, with significant differences (Table 2). Unfortunately, the heterogeneity is too large, which reduces the reliability of the data. Therefore, we carried out the next analysis of various subgroups.

**TABLE 2:** The results of all outcomes: all participants.

Outcomes		Number of study	Risk ratio or mean difference (95% CI)	*P*-value	*P* for heterogeneity	*I*^2^ (%)	Quality
BMD	Spine	9	0.20 [0.09, 0.31]	<0.0001	<0.00001	86	Low
	Femur	6	0.05 [−0.01, 0.12]	0.09	<0.00001	87	Low
	Hip	3	0.15 [−0.23, 0.54]	0.01	0.61	0	Low
VAS		4	−0.64 [−0.91, −0.38]	<0.00001	0.06	60	Low
BBS		5	5.05 [3.17, 6.93]	<0.00001	0.004	74	Low
Ca		3	0.02 [−0.06, 0.10]	0.63	0.11	55	Low
P		3	0.03 [−0.01, 0.06]	0.19	0.97	0	Low
ALP		4	1.63 [−2.15, 5.40]	0.40	0.09	53	Low
BGP		2	0.81 [0.64, 0.99]	<0.00001	0.39	0	Low

ALP, alkaline phosphatase; BBS, Berg balance scale; BGP, bone gla protein; BMD, bone mineral density; Ca, serum calcium; P, serum phosphorus; RCTs, randomized controlled trials; VAS, visual analog scale.

**FIGURE 3:**
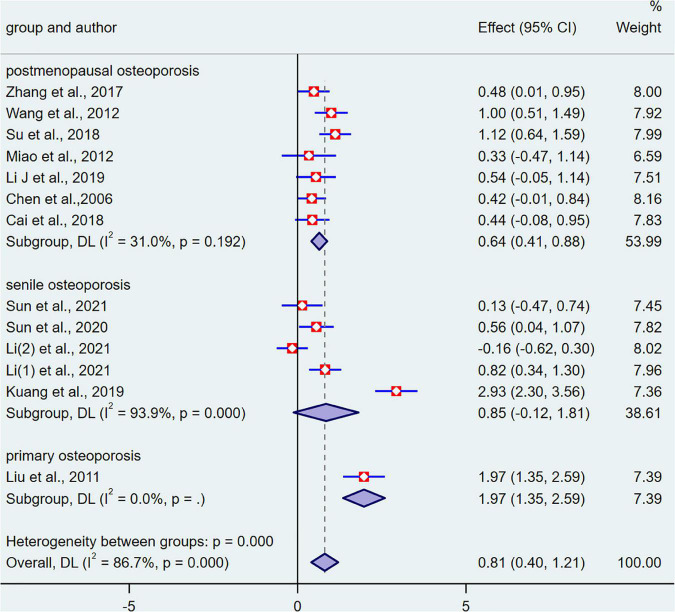
The bone mineral density (BMD) results of all participants for three subgroups: the control group *vs.* the intervention group.

First, we divided into three groups according to the type of OP (postmenopausal osteoporosis, senile osteoporosis, and primary osteoporosis) for subgroup analysis (Figure 3). Unfortunately, the heterogeneity we obtained was still very high. We then divided into two subgroups based on different treatments: BDJ exercise *vs.* no treatment (subgroup 1), BDJ exercise *vs.* BDJ exercise + conventional treatment (subgroup 2). Meanwhile, subgroup 1 and subgroup 2 were divided into subgroups 3–7, according to the type of OP. Within these subgroups, we assessed primary and secondary outcomes for all participants separately (Table 3). For postmenopausal osteoporosis, BDJ exercise alone and BDJ exercise combined with conventional treatment can improve the BMD of lumbar spine and VAS. BDJ exercise alone can influence Ca, ALP. BDJ exercise combined with conventional treatment improved balance (BBS), and influenced BGP. For senile osteoporosis, BDJ exercise alone and BDJ exercise combined with conventional treatment can improve balance (BBS). BDJ exercise combined with conventional treatment can improve the BMD of hip and relieve pain (VAS). For primary osteoporosis, BDJ exercise combined with conventional treatment can improve the BMD of lumbar spine and femoral neck. However, BDJ exercise did not influence serum Ca and serum P level.

**TABLE 3:** The results of all outcomes for five subgroups: the control group vs. the intervention group.

Group	Subgroups	Categories	Outcomes		Number of study	Risk ratio or mean difference (95% CI)	*P*-value	*P* for heterogeneity	*I*^2^ (%)	Quality
BDJ exercise *vs*. no treatment	PMOP	Primary outcome	BMD	Spine	4	0.60 [0.30, 0.90]	<0.0001	0.31	17	Low
				Femur	1	0.01 [−0.06, 0.08]	0.77			Low
				Hip	2	0.57 [−0.14, 1.27]	0.11	0.51	0	Low
		Secondary outcomes	VAS		1	−1.71 [−1.75, −1.67]	<0.00001			Low
			BBS		–					
			Ca		1	0.13 [0.01, 0.26]	0.04			Low
			P		1	0.03 [−0.07, 0.13]	0.57			Low
			ALP		2	5.25 [1.50, 9.01]	0.006	0.72	0	Low
			BGP		1	2.26 [−1.07, 5.59]	0.18			Low
	SOP	Primary outcome	BMD	Spine	2	0.32 [−0.62, 1.27]	0.5	0.004	88	Low
				Femur	–					
				Hip	2	0.57 [−0.14, 1.27]	0.11	0.51	0	Low
		Secondary outcomes	VAS		–					
			BBS		2	3.42 [1.99, 4.85]	0.004	0.1	63	Low
			Ca		–					
			P		–					
			ALP		–					
			BGP		–					
BDJ exercise *vs.* BDJ exercise + conventional treatment	PMOP	Primary outcome	BMD	Spine	3	0.68 [0.25, 1.11]	0.002	0.09	58	Low
				Femur	1	0.00 [−0.46, 0.46]	1			Low
				Hip	–					
		Secondary outcomes	VAS		2	−1.37 [−1.40, −1.35]	<0.00001	<0.00001	97	Low
			BBS		1	4.47 [0.12, 8.82]	0.04			Low
			Ca		2	−0.02 [−0.07, 0.04]	0.58	0.85	0	Low
			P		2	0.02 [−0.02, 0.07]	0.24	0.81	0	Low
			ALP		2	−0.96 [−4.06, 2.13]	0.54	0.99	0	Low
			BGP		1	0.81 [0.63, 0.99]	<0.00001			Low
	SOP	Primary outcome	BMD	Spine	3	1.19 [−0.42, 2.80]	0.15	<0.00001	96	Low
				Femur	2	0.01 [−0.00, 0.03]	0.14	0.27	18	Low
				Hip	1	0.01 [0.00, 0.02]	0.03			Low
		Secondary outcomes	VAS		3	−0.67 [−1.07, −0.27]	0.001	0.02	73	Low
			BBS		2	5.95 [4.85, 7.04]	<0.0001	0.03	80	Low
			Ca		–					
			P		–					
			ALP		–					
			BGP		–					
	POP	Primary outcome	BMD	Spine	1	0.21 [0.16, 0.26]	<0.00001			Low
				Femur	1	0.23 [0.17, 0.28]	<0.00001			Low
				Hip	–					
		Secondary outcomes	VAS		–					
			BBS		–					
			Ca		–					
			P		–					
			ALP		–					
			BGP		–					

PMOP, postmenopausal osteoporosis; SOP, senile osteoporosis; POP, primary osteoporosis; ALP, alkaline phosphatase; BBS, Berg balance scale; BGP, bone gla protein; BMD, bone mineral density; Ca, serum calcium; P, serum phosphorus; RCTs, randomized controlled trials; VAS, visual analog scale.

### Funnel plot analysis

**FIGURE 4:**
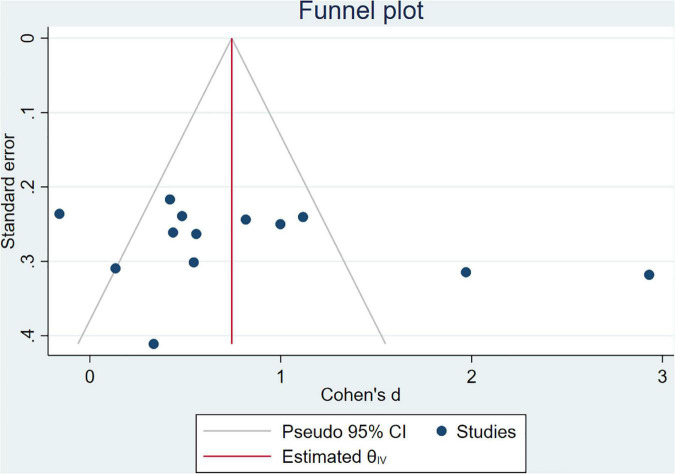
Funnel plot analysis: publication bias.

By analyzing the full cases of BMD in patients, there was no evidence of publication bias according to the Begg’s test (*P* = 0.428) and Egger’s test (*P* = 0.200) for the meta-analysis (Figure 4).

### Overall quality of evidence by GRADE

We scored the overall quality of the available evidence using GRADE pro^2^ (Supplementary Figure 1). The quality of evidence for all outcomes was downgraded to “low,” mainly due to high risk of performance bias and imprecision (small total number of events or sample size).

### Trial sequential analysis

We performed TSA on the BMD of the total patients and two subgroups divided by different interventions (BDJ exercise alone, and BDJ exercise combined with conventional treatment). The required information size (RIS) of the total patients was 1,373 (Supplementary Figure 2), and the RIS of the BDJ exercise alone intervention was 3,794 (Supplementary Figure 3). The RIS of BDJ exercise combined with conventional treatment intervention was 1,242 (Supplementary Figure 4). RIS was estimated based on the following statistical indicators: Type I error probability (α = 0.05), Type II error probability (β = 0.2), mean difference and variance data from this meta-analysis. The results of TSA in the three cases showed that the cumulative Z value crossed the traditional threshold and also crossed the TSA threshold, indicating that a positive conclusion had been obtained before the expected amount of information had been reached, that is, BDJ exercise had a positive effect on BMD in patients with OP, whether it was BDJ exercise alone intervention or BDJ exercise combined with conventional treatment, the evidence was clear. Although the TSA analysis supported the results of the meta-analysis, the 13 included RCTs were all rated as low-quality, and the impact of the methodological quality of the trial itself on the results of the meta-analysis cannot be ignored. Therefore, we still need high-quality research to support this evidence.

## The possible mechanism of Baduanjin exercise

**FIGURE 5:**
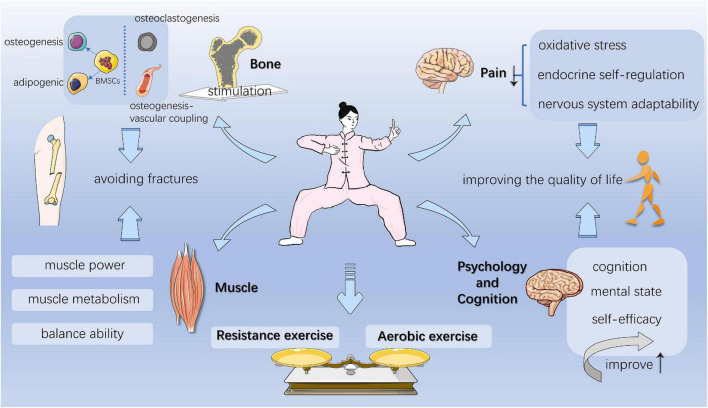
The potential mechanism of Baduanjin (BDJ) exercise.

Studies have shown that in the RCTs conducted, the overall compliance rate is satisfactory, and no major adverse events are found, such as falls and injuries, indicating that BDJ exercise is safe and feasible in the elderly population (16–18). From the perspective of physiological effects, BDJ exercise is very advantageous in the treatment of OP. It strengthens tendons and bones, and improves bone strength (19). It can systematically mobilize active joints and muscles, improve the balance ability, enhance cardiorespiratory function, regulate body and mind at the same time, and finally achieve the unity of body and mind (6, 14). Therefore, it is necessary to study its effective mechanism (Figure 5).

### Baduanjin exercise can produce stress stimulation on bones, and play a role in regulating bone mass

The ability of exercise to improve BMD appears to be well proved (20). The main outcome measure of this meta-analysis was BMD. All the included studies showed that BDJ exercise can effectively improve BMD, and some studies have mentioned that it has a good promoting effect on different parts (lumbar spine, femoral neck, trochanter, Ward triangle, etc.). There is evidence that aerobic exercise can increase BMD, while the combination of resistance training and balance training can prevent the risk of falls and fractures in the elderly, and studies have pointed out that the best type of exercise to improve BMD in the spine and hip is progressive resistance training (21). BDJ exercise has the characteristics of both aerobic exercise and resistance exercise.

Bone is a living tissue that responds to mechanical stimulation. For older adults with low bone mass, exercise is an effective osteogenic therapy (22). The process of BDJ exercise generates a variety of mechanical loads, such as tension, compression and fluid shear stresses, which has beneficial effects on reducing bone loss, increasing bone strength and preventing osteoporosis in the elderly (23). It almost can act directly or indirectly on all types of bone cells and affect many aspects of bone remodeling. Osteogenic and osteolytic activities are two significant physiopathological processes in osteoporosis, and bone tissue cells are a mechanical sensor capable of converting musculoskeletal-derived mechanical stimulus inputs into biological signal outputs (24).

#### Osteogenic activity

In osteogenic activity, the Wnt signaling pathway is a central pathway for osteoblast differentiation and function (25). Exercise-induced mechanical stress stimuli regulate bone remodeling by affecting osteoblasts (26, 27). Osteoblasts can produce non-collagenous proteins, including osteocalcin (a small vitamin K-dependent calcium-binding protein that targets bone) and alkaline phosphatase, which is essential for mineral deposition (28). Meanwhile alkaline phosphatase is involved in the transformation of endochondral ossification (which produces most bone) into a cartilage model that resembles the shape of bone during osteogenesis (29). In this analysis, it was also precisely obtained that the BDJ exercise improved serum osteocalcin and alkaline phosphatase levels, reflecting to some extent that BDJ exercise can affect osteogenic activity. Osteoblast-derived sclerostin regulates osteoblast and osteoclast activity. Sclerostin is a downstream mediator of the skeletal response to the mechanical environment and is able to inhibit the Wnt/β-catenin signaling pathway in osteoblasts (30). A cross-sectional observational study (25) showed that serum sclerostin levels increased with age in both healthy men and premenopausal women aged 19–64 years, were positively correlated with BMI and bone mineral content, and negatively correlated with osteocalcin (a marker of bone formation) and calcium levels. It is still a factor to worth exploring how serum sclerostin levels change after Baduanjin exercise. Whole-body vibration (WBV) is commonly studied in animals to mimic the changes brought about by exercise *in vivo*. WBV therapy can mimic the mechanical signals normally generated by low-intensity activities (e.g., postural muscle contractions) and is also considered an effective treatment for OP (31, 32), which also exerts positive osteogenic effects through osteoblasts and the Wnt-β-catenin signaling pathway (33). However, BDJ exercise could not be realized in animal studies due to its own specificity, and WBV therapy reference is informative.

#### Osteogenesis-lipogenesis and osteogenesis-vascular coupling activity

In addition, a strong body of evidence suggests that mechanical stress stimulation directly induced by exercise can affect the osteogenic and lipogenic differentiation processes of bone mesenchymal stem cells (BMSCs) (34). Mechanical loading has a positive effect on osteogenic differentiation of BMSCs, mainly by promoting osteogenic differentiation and inhibiting lipogenic differentiation, which may also be one of the main reasons for the prevention of OP by BDJ exercise. Bone is a highly vascularized tissue, and exercise mechanical loading stimulates an angiogenic osteogenic response in the skeleton (35). Angiogenesis plays an important role in bone formation and bone remodeling, and skeletal blood supply is critical for maintaining bone density and bone structure. HIF-1α has recently been implicated as a negative regulator of the osteoblast response to mechanical loading. The response of osteoblasts in oxygen tension is directed through the activity of hypoxia-inducible factor (HIF), which activates gene transcription in response to low oxygen levels (36). The expression of HIF-1α in osteoblast cell lines is required for angiogenesis (37), and the mediator role of HIF-1α in osteoblast-angiogenic coupling cannot be ignored. It is well known that BDJ exercise is a traditional low aerobic exercise, and it is feasible to study its effect on bone mass and bone strength by cutting from the perspective of HIF-1α.

#### Osteoclastic activity

Overall, osteoclastic activity tends to be less studied in osteoporosis as a whole, but its role in exercise should not be overlooked. Exercise can inhibit osteoclastogenesis and bone resorption through BMSCs, osteoblasts, and the OPG/RANKL pathway secreted by pro-inflammatory cytokines (38). As the skeletal load generated after exercise leads to microdamage (which has been shown to trigger bone remodeling), apoptosis occurs in osteoblasts located near the site of microdamage, which is associated with increased bone remodeling due to increased RANKL production and increased osteoclast formation (39).

In conclusion, there is a strong tendency to infer a beneficial effect of BDJ exercise on osteoporosis based on the currently available studies, even if we do not have more direct evidence to prove it. This beneficial effect is reflected not only in osteogenic and osteolytic activities, but also in new research hotspots, such as lipid-bone metabolism, and osteogenic-vascular coupling. Even so, not all forms of exercise have a positive effect on bone mass. For example, no-load exercise (swimming, cycling, etc.) had no effect on bone mass, while walking or running had limited positive effects (20). In addition, there is a near gap in direct studies on stress stimulation by BDJ exercise in humans, and future research would be better focused on understanding how osteoblasts sense mechanical load after BDJ exercise and translate mechanical signals into signals that regulate bone mass.

### Baduanjin exercise can improve muscle strength and metabolism, and improve balance ability

The relationship between muscle and bone is always complementary and inseparable, muscle contraction and relaxation may also stimulate osteogenesis, and men with strong muscle strength also have stronger bone strength (40). Many studies have shown that exercise can enhance muscle strength. From the perspective of its own movements, the continuous half-squat posture of BDJ exercise will constantly challenge the postural stability of lower limbs and the body balance. Specifically, the feet should be consistent with the ground, and cannot cross the boundary of the shoulder width (similar to the oval area) (41). Some studies have focused the effect of BDJ exercise on the lower limbs muscle strength on the vastus medialis muscle strength. After BDJ exercise, the root mean square (RMS), integrated electromyogram (IEMG), and average electromyographic (AEMG) of the vastus medialis muscle elevated, the vastus lateralis muscle did not change significantly (42). It may be that the intervention time is only 16 weeks, and the time span is not long enough. In addition, some studies have shown that not only the muscle strength of the lower limbs, but also the grip strength has been significantly improved (43), so BDJ exercise has a certain positive effect on the improvement of the muscle strength of the whole body.

Exercise is one of the most powerful regulators of metabolism, and BDJ exercise may be more beneficial to the body than other forms of exercise, because it requires lower metabolic demands. Studies have shown that in the process of improving muscle strength, muscles involved in exercise increase local capillaries, and oxygen supply also needs to increase, HIF-1 inhibits skeletal muscle mTOR activity under low energy/hypoxia conditions, and is essential for restoring mTOR signaling when cells recover from hypoxia (44). In addition, exercise increases whole-body energy expenditure and induces lipolysis, and improves systemic metabolism. Studies have shown that BDJ exercise can significantly increase serum HDL-C level, and reduce TC, TG and LDL-C in middle-aged and elderly people (45). Long-term exercise can activate lipoprotein (a rate-limiting enzyme that causes the breakdown of TG and determines the amount of fatty acids in skeletal muscle), thereby accelerating lipid metabolism and improving blood lipid levels (46). We can focus on this in future research to further explore more in-depth mechanisms.

Of course, the actual goal of exercise intervention is not only to maintain bone mass, but also to reduce the incidence of osteoporotic fractures. Falls are one of the most common factors affecting the quality of life in the elderly. Muscle strength and balance are important components of good health, which provides information on the ability of older adults to reduce the risk of falls. Studies have shown that BDJ exercise can reduce the fear of falling and improve balance (47, 48). Physical flexibility is closely related to balance ability, and the increase of physical flexibility can improve the coordination ability of the body, thereby improving the balance of the body, which is a key factor in preventing falls (49). Experiments have found that BDJ exercise can significantly improve body flexibility (50). In addition, studies have shown that BDJ exercise can significantly improve spatial gait parameters such as stride length, walking speed, and stride frequency, which are also closely related to lower limb muscle strength and balance (11). Not only in osteoporosis, but also in other diseases, BDJ exercise is effective in improving balance, leg muscle strength and flexibility in chronic stroke patients. For patients with Parkinson’s disease, BDJ exercise can also improve balance and lower extremity strength, and reduce the risk and rate of falls (51).

### Baduanjin exercise can improve oxidative stress and neuro-endocrine regulation, and relieve pain symptoms

The efficacy of exercise and the level of evidence vary in different diseases, but exercise has direct and indirect benefits for most patients with chronic pain (52). Pain is the most expressive clinical symptom in patients with OP and cannot be ignored. Combined with other studies, there is strong evidence that BDJ exercise can reduce pain in patients with OP. BDJ exercise is a full-body exercise, and all forms of BDJ exercise combine rhythmic movements or fixed body postures with deep breathing techniques to reduce the appearance of pain, which may be closely related to the matching of breathing rate. Some studies have pointed out that during the exercise, the spine, especially the neck, can perform movements such as twisting, extension and forward flexion, which can fully relax the muscles of the neck and lower limbs, reduce pain-causing stimulation, and improve pain behavior and physical activity tolerance (34).

One mechanism may be that exercise improves aerobic capacity, and protects against oxidative stress. Regular exercise helps protect the body from oxidative stress, and may vary with the duration and intensity of exercise (53). As a health qigong, BDJ has a heart rate of 90–110 beats/min during exercise, which is a low-intensity aerobic exercise (54). Some studies have pointed out that BDJ exercise is a safe and feasible option for the treatment of knee osteoarthritis (KOA). It can reduce pain, stiffness, enhance the strength of knee extensors and flexors, and improve the aerobic capacity of patients (55). Similarly, quadriceps strengthening exercise (QSE) combined with BDJ exercise significantly reduced the pain degree of KOA patients, and the persistence of prognosis improvement was relatively better (56). Exercises like BDJ that combine mind-body and physical exercises can relieve pain symptoms and produce stronger therapeutic effects than exercise alone (57). BDJ exercise may lead to a significant decrease in Malondialdehyde (MDA) levels (58), which may be related to the production of superoxide dismutase (SOD), sensitivity to oxidants, antioxidant enzyme activities, and increases in antioxidant levels. A reduction in MDA levels may be beneficial for body function (58).

Factors such as endocrine self-regulation and central nervous system adaptability may play a role in the mechanism which BDJ exercise improves pain. Studies have shown that BDJ exercise can enhance the cognitive function of the elderly, which may also help relieve pain. Exercise can simultaneously modulate the opioid antihypertensive pathway and resting-state functional connectivity (rsFC) and blood inflammatory markers of the brain system. BDJ exercise can increase serum programmed death-1 (PD-1) concentration and reduce serum Interferon-γ (INF-γ) concentration (59), and also reduce the rsFC between VTA and MOPFC, which were significantly correlated with the corresponding PD-1 at baseline, suggesting that PD-1 may play a regulatory role in post-exercise pain relief through the regulation of VTA, MOPFC, and rsFC (57). A previous study suggested that disruption within the default mode network (DMN) may underlie chronic pain-related cognitive and behavioral impairments (60), and BDJ exercise increased the rsFC between the left periaqueductal gray (PAG) and right thalamus, which may reflect an increase in attentional control in pain regulation (57).

### Baduanjin exercise can improve cognitive dysfunction and psychological state, and has a positive effect on improving the quality of life

Baduanjin exercise is the most studied in terms of improving cognition and psychological state, both in terms of clinical efficacy and mechanism of action, and its mechanism is also most clearly explained. BDJ exercise is more suitable for people with cognitive impairment because of its simplicity and easy learning, and may be an effective method to prevent cognitive decline in the elderly (61, 62). There is evidence that 12-week BDJ exercise can significantly increase insula, medial temporal lobe and putamen gray matter volume (GMV), amplitude of low frequency fluctuation (ALFF), and medial prefrontal cortex (mPFC), especially improvements in memory were found to positively correlate with increased GMV in subjects’ left putamen and hippocampus (63, 64). These regions are precisely the key to memory and cognition, especially GMV is relatively important for maintaining memory function (61). The appearance of these phenomena is not accidental, nor is it affected by the intervention time. A study found that the right GMV also increased in the 24-week BDJ exercise (65). Therefore, a series of studies suggest that BDJ exercise may be a potentially beneficial intervention for improving the attention of older adults with mild cognitive impairment (66).

Mental states and functions are linked to the functioning of cognitive control networks. A large body of evidence indicates that BDJ exercise also has a very strong effect on the improvement of mental state and function (67). BDJ exercise affects the mood of the patients during the training process, especially helps to reduce negative emotions such as depression and anxiety. For example, in the section concentration enhancement and breathing control can strengthen the functional connection of cognitive control network, while breathing regulation can regulate the autonomic nervous system and endocrine system by increasing the oxygen consumption, carbon dioxide production, cardiac output and plasticity of the autonomic nervous system (68, 69). These results lead to a calm and stable emotional state that restores homeostasis.

Baduanjin exercise in daily life also provides a new social channel and improves self-efficacy (48). A 16-week community-based BDJ exercise intervention has potential benefits for physical, cognitive, and mental health in frail older adults, and may reduce symptoms of frailty and even reverse the state of frailty (18). Chinese Qigong, for example, BDJ exercise can enhance the self-efficacy of participants, thereby reducing depressive symptoms (70). All practitioners reported significant increases in their subjective calmness and perceived physical activation, concentration, and subjective vitality (71). With the gradual improvement of balance and coordination, the subjects’ motor function and self-confidence correspondingly increased (48). Neurobiological pathways have been proposed to explain the antidepressant effect of Chinese Qigong (69). There are three ways (72): up-regulation of monoamines neurotransmitter-like, stimulation of the hypothalamic-pituitary-adrenal axis, and upregulation of brain-derived neurokines, but they need to be further tested. It is hypothesized that (69), BDJ exercise can reduce stress signals sent to the hippocampus and amygdala of the limbic system, reduce the secretion of corticotropin-releasing factor in the periventricular hypothalamus and the release of corticotropin in the anterior pituitary, and then downregulate the hyperactivity of the HPA axis in depressed individuals. Even so, we still need to devote more energy to elucidating the mechanism.

## Discussion

This systematic review explores the efficacy of BDJ exercise in the treatment of OP by analyzing 13 RCTs with 919 participants. The BDJ exercise differs mainly in the frequency, duration, and settings of the intervention, and none of the included studies evaluated the sustained effects after the intervention in this review. We find that BDJ exercise has a positive effect on maintaining BMD, relieving pain, and holding balance in patients with OP, either as a monotherapy or combination with conventional treatment. We assessed the evidence from the GRADE approach, but were unable to draw definitive conclusions due to the low quality of the included trials and the fact that these results are from individual trials. In addition, the heterogeneity of this meta-analysis is high, and we have avoided its negative effects as much as possible. Therefore, high-quality research can improve the accuracy of our assessment of BDJ exercise.

The same themes are reported in the two previous meta-analyses (Table 4). The advantages of our meta-analysis are as followed. First, our analysis is more rigorous and of higher quality, we strictly follow the “PICOS” principle for study screening, and it is worth mentioning that we evaluate the intervention behavior of the control group, in that it limits the scope of conventional treatment (guideline-recognized drugs), which is not achieved in previous meta-analyses. Second, as the most recent and most comprehensively updated meta-analysis, the present study further strengthens the results of previous meta-analyses, in which BDJ exercise has a beneficial effect in improving symptoms in patients with OP. Third, we employ TSA to estimate effects more conservatively, focusing on more representative and specific results, which may adequately describe the effects of BDJ exercise in patients with OP and strengthen the existing evidence. In addition, we also preliminarily summarize the potential mechanism of BDJ exercise in the treatment of OP.

**TABLE 4:** Comparison with other previous meta-analyses.

Author	Li et al. (88)	Yu (89)	The present meta-analysis
Number of RCTs	15	4	13
Participants	Osteoporosis	Senile osteoporosis	Osteoporosis
Search strategy until	January 2019	January 2020	April 2022
Protocol registered	**–**	**–**	Applied
Trial sequential analysis	**–**	**–**	Applied
Outcomes	BMD (lumbar spine, femoral neck), VAS, BGP, ALP, and Ca	BMD (lumbar spine, femoral neck, Ward’s triangle, and greater trochanter), ALP	BMD (lumbar spine, femoral neck, hip), VAS, BBS, Ca, P, ALP, and BGP

ALP, alkaline phosphatase; BBS, Berg balance scale; BGP, bone gla protein; BMD, bone mineral density; Ca, serum calcium; P, serum phosphorus; RCTs, randomized controlled trials; VAS, visual analog scale.

However, several limitations are worth considering. Due to limited reports, all the participants are from China, and the sample size is generally small. We have to admit that the extrapolation and reliability will be compromised. Further trials are needed in a larger comprehensive population to demonstrate the effectiveness of BDJ exercise in improving BMD and clinical symptoms. In addition, we believe that long-term tracking of the subjects’ follow-up exercise is required, as it may take a long time, even years, to show the benefit of BDJ exercise. However, there was no long-term follow-up in the currently included studies. In addition, BDJ exercise is based on traditional exercise, which cannot be blind to researchers and patients, thus possibly introducing bias. At the same time, the included RCTs were conducted in different populations and in different clinical settings, therefore, there is a potential risk of heterogeneity. Besides, the non-standardized methodology and the small number of included trials may lead to overestimation of the overall efficacy of BDJ exercise. Therefore, high-quality studies are needed to confirm the efficacy of BDJ exercise on OP.

On the whole, BDJ exercise is a healthy way worthy of attention and promotion. When it comes to traditional Chinese exercise, it is easy to compare it with Tai Chi. Compared with Tai Chi involving at least twenty-four movements, BDJ exercise only contains eight independent and smooth movements. This determines that BDJ exercise has fewer physical and cognitive requirements, and is easier to learn and practice. However, further research is needed on the stress stimulation of two exercises on bones. In addition, some studies (20, 73, 74) mentioned that all forms of exercise can positively affect bone mass, but it is unclear which type of exercise is the best way to build bone mass. At the same time, not all forms of exercise positively affect bone mass. For example, no-load exercise, such as swimming and cycling, had no effect on bone mass, while walking or running had a limited positive effect. However, the shining point of BDJ exercise is that it combines the characteristics of resistance exercise and aerobic exercise, and the stimulation of bone mass is gentle and lasting. Other than that, few other types of exercise involve breathing and meditation in the process. All in all, BDJ exercise has unique and incomparable advantages.

In addition, we preliminarily infer the therapeutic mechanism of BDJ exercise in the treatment of OP by summarizing the existing evidence. Each section of BDJ exercise can take into account the clinical manifestations of OP, which is reflected in the beneficial effects on bone, muscle, pain, psychology, and cognition. Although we have preliminarily sorted out and revealed relevant evidence, there is still a lack of direct confirmation of high-level evidence-based medicine, and the multi-pathway mechanism of BDJ exercise still needs to be further studied.

## Conclusion

This meta-analysis presents some insights from research and practice, and initially summarizes the mechanism of BDJ exercise to help further understand the benefits of it on the health and well-being of the elderly. The review indicates that BDJ exercise may have a certain effect on improving BMD, relieving pain symptoms and improving balance ability in patients with OP. Through our preliminary exploration of the mechanism, we believe that BDJ exercise is an ideal aerobic training activity, a combination of resistance exercise and strength training. In conclusion, BDJ exercise is an effective, feasible and safe exercise program for the elderly with OP.

## Data availability statement

The original contributions presented in the study are included in the article/Supplementary material, further inquiries can be directed to the corresponding authors.

## Author contributions

CS, BQ, YZ, XW, and LZ chose the topic. CS, MC, and ZJ collected the data. CS, BQ, and XH performed the analysis. YZ, XW, and LZ made final decisions. All authors contributed to writing the manuscript and approved the submitted version.

## Footnotes

1
http://www.crd.york.ac.uk/PROSPERO

2
https://gdt.gradepro.org
