# Supplementary material for: Baduanjin exercise: A potential promising therapy toward osteoporosis
Source: Front Med (Lausanne). 2022 Aug 3;9:935961. doi: 10.3389/fmed.2022.935961 (PMC9381703; doi:10.3389/fmed.2022.935961)
Supplement: Supplementary file 1 [file Data_Sheet_1.docx]

Supplementary Material

# Supplementary Figures

**
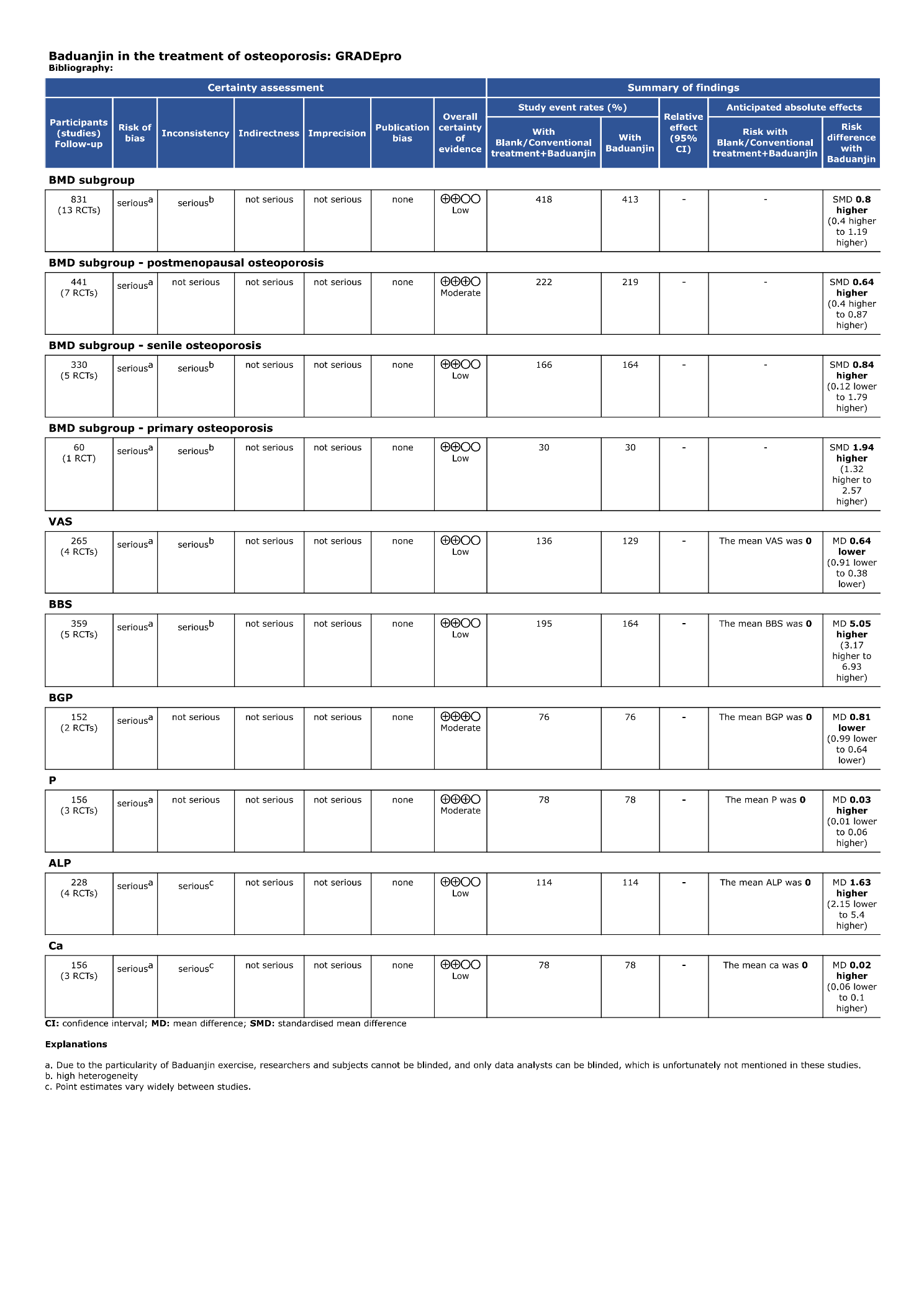
**

Supplementary Figure 1 Overall quality of evidence by GRADE


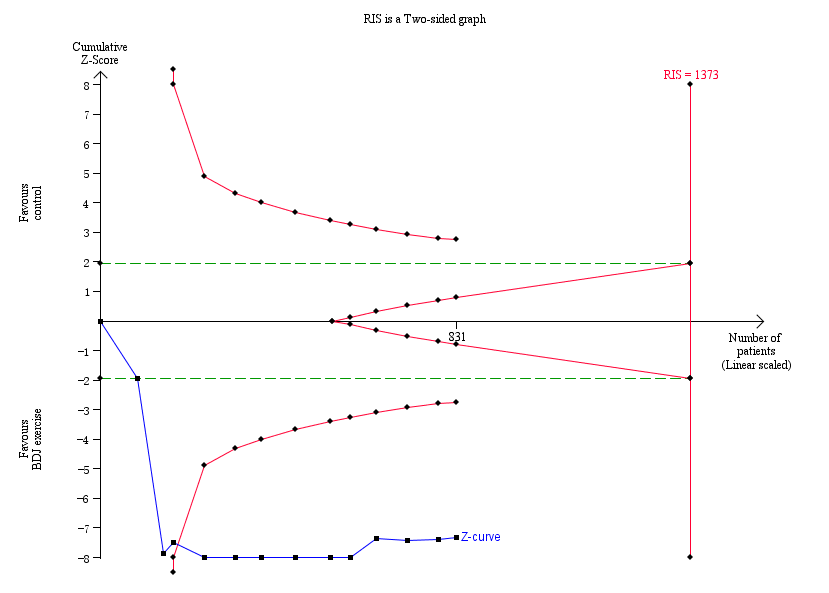


Supplementary Figure 2 TSA for BMD value (all participants) with an α of 5% (two-sided) and β of 20%

**Notes:** The required information size was calculated as 1373. Z curve has across-trial sequential monitoring boundary for benefit (all participants).


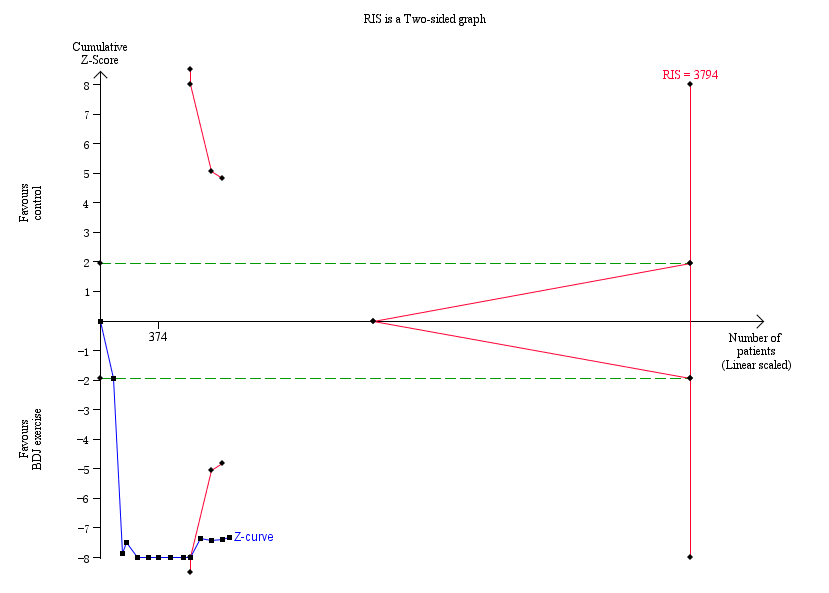


Supplementary Figure 3 TSA for BMD value (blank vs BDJ exercise intervention) with an α of 5% (two-sided) and β of 20%

**Notes:** The required information size was calculated as 3794. Z curve has across-trial sequential monitoring boundary for benefit (blank vs BDJ exercise intervention).


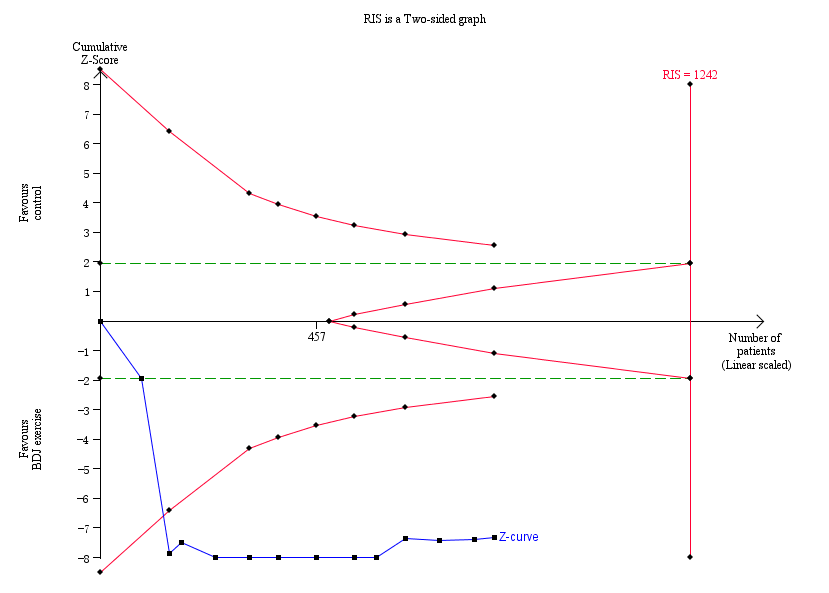


Supplementary Figure 4 TSA for BMD value (conventional treatment vs BDJ exercise combined with conventional treatment) with an α of 5% (two-sided) and β of 20%

**Notes:** The required information size was calculated as 1242. Z curve has across-trial sequential monitoring boundary for benefit (BDJ exercise combined with conventional treatment).
